# Radiosynthesis and Early Evaluation of a Positron Emission Tomography Imaging Probe [^18^F]AGAL Targeting Alpha-Galactosidase A Enzyme for Fabry Disease

**DOI:** 10.3390/molecules28207144

**Published:** 2023-10-18

**Authors:** Talakad G. Lohith, Charalambos Kaittanis, Anthony P. Belanger, Shin Hye Ahn, Phil Sandoval, Lawrence Cohen, Girija Rajarshi, Wanida Ruangsiriluk, Rizwana Islam, Christopher T. Winkelmann, Paul McQuade

**Affiliations:** 1 Takeda Pharmaceutical Co., Ltd., Cambridge, MA 02142, USA; bambos.kaittanis@takeda.com (C.K.); phil.sandoval@takeda.com (P.S.); lawrence.cohen@takeda.com (L.C.); girijarajarshi@gmail.com (G.R.); ruangw29@hotmail.com (W.R.); rislam@post.harvard.edu (R.I.); christopher.winkelmann@takeda.com (C.T.W.); paul.mcquade@takeda.com (P.M.); 2 Molecular Cancer Imaging Facility, Dana Farber Cancer Institute, Boston, MA 02210, USA; anthony_belanger@dfci.harvard.edu (A.P.B.); shinh_ahn@dfci.harvard.edu (S.H.A.)

**Keywords:** Fabry disease, alpha-galactosidase A, positron emission tomography, fluorine-18, radiolabeling, AGAL

## Abstract

Success of gene therapy relies on the durable expression and activity of transgene in target tissues. In vivo molecular imaging approaches using positron emission tomography (PET) can non-invasively measure magnitude, location, and durability of transgene expression via direct transgene or indirect reporter gene imaging in target tissues, providing the most proximal PK/PD biomarker for gene therapy trials. Herein, we report the radiosynthesis of a novel PET tracer [^18^F]AGAL, targeting alpha galactosidase A (α-GAL), a lysosomal enzyme deficient in Fabry disease, and evaluation of its selectivity, specificity, and pharmacokinetic properties in vitro. [^18^F]AGAL was synthesized via a Cu-catalyzed click reaction between fluorinated pentyne and an aziridine-based galactopyranose precursor with a high yield of 110 mCi, high radiochemical purity of >97% and molar activity of 6 Ci/µmol. The fluorinated AGAL probe showed high α-GAL affinity with *IC*_50_ of 30 nM, high pharmacological selectivity (≥50% inhibition on >160 proteins), and suitable pharmacokinetic properties (moderate to low clearance and stability in plasma across species). In vivo [^18^F]AGAL PET imaging in mice showed high uptake in peripheral organs with rapid renal clearance. These promising results encourage further development of this PET tracer for in vivo imaging of α-GAL expression in target tissues affected by Fabry disease.

## 1. Introduction

Imaging probes labeled with positron emitting radioisotopes with short half-lives have the distinct advantage of characterizing target proteins in vivo in living systems. Over the last few decades, multiple positron emission tomography (PET) tracers have been developed, targeting specific cell surface receptors and intracellular enzymes affected in neurological, oncological, and cardiovascular disorders. However, there are no PET tracers targeting the intracellular lysosomal enzymes that are primarily affected in lysosomal storage disorders (LSDs). Fabry disease is a rare X-linked LSD which results from a dysfunctional alpha-galactosidase A (α-GAL) enzyme leading to accumulation of glycosphingolipids in cells of the heart, kidneys, nervous system, and other organs [1]. Current standard-of-care treatment for Fabry patients includes enzyme replacement therapy (ERT), which involves intravenous administration of recombinant α-GAL enzyme and monitoring α-GAL serum activity and concentrations as proxy measures of effectiveness of ERT in clinical studies. Since the replacement enzyme is a large molecule with limited access to the intracellular compartment, particularly in key cells of the heart and kidneys, and concentrations fluctuate due to biweekly treatment, it is posited that emerging gene therapies would provide necessary high intracellular levels and sustained exposure to all of the key target tissues [2]. Success of gene therapy for Fabry disease therefore depends on the reliable expression of active α-GAL in the intracellular compartment of the targeted tissues. In this regard, a PET tracer that can accurately reflect the magnitude, duration, and location of the intracellular α-GAL expression and/or activity in vivo would be highly valuable as a pharmacokinetic and pharmacodynamic (PK/PD) biomarker for disease monitoring and treatment response in a gene therapy trial.

Currently established measures of α-GAL activity include in vitro assays that use fluorogenic substrates such as 4-methylumbelliferyl-α-d-galactopyranoside (4-MUGA), which has high selectivity and specificity to the α-GAL enzyme. However, those molecules are not facile for labeling with PET radioisotopes. In 2014, Willems et al. reported a fluorescent Bodipy-aziridine molecule (compound **3**), an activity-based probe (ABP) that binds covalently with high affinity to recombinant AGAL enzyme and enables the visualization of the endogenous activity of AGAL enzyme in fibroblast cell extracts [3]. Based on compound **3** published in Willems et al., herein we report radiosynthesis of [^18^F]AGAL, a PET probe targeting α-GAL, from a facile aziridine-based galactopyranose precursor molecule and characterize it in vitro for selectivity, specificity, and other physicochemical properties, as well as test for in vivo biodistribution in control mice via PET imaging.

## 2. Results and Discussion

### 2.1. Synthesis of Radiolabeling Precursors and Reference Standards

**Figure 1:**
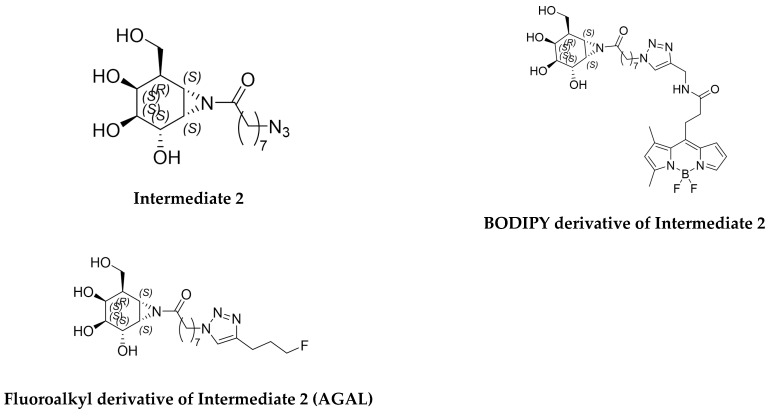
AGAL precursor molecule (Intermediate 2), BODIPY derivative of Intermediate 2, and fluoroalkyl derivative of Intermediate 2 (AGAL).

Multi-step syntheses were carried out to prepare three compounds—AGAL precursor molecule (Intermediate 2), fluoroalkyl derivative of Intermediate 2 (AGAL), and BODIPY derivative of Intermediate 2—at MedChem Imaging, Inc. (Boston, MA, USA)—following the literature procedures [3,4,5,6] with minor modifications to scale the reactions to 100, 50, and 20 mg, respectively. All compounds were characterized with ^1^H NMR spectroscopy and LC–MS for the structure and purity (Figure 1).

### 2.2. Synthesis of [^18^F]AGAL

**Figure 2:**
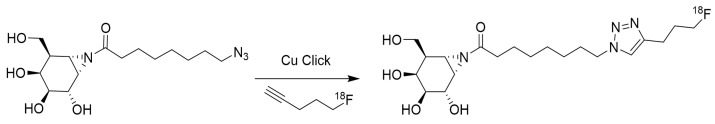
Radiosynthesis of [^18^F]AGAL PET tracer using Cu-mediated azide/alkyne click chemistry.

**Figure 3:**
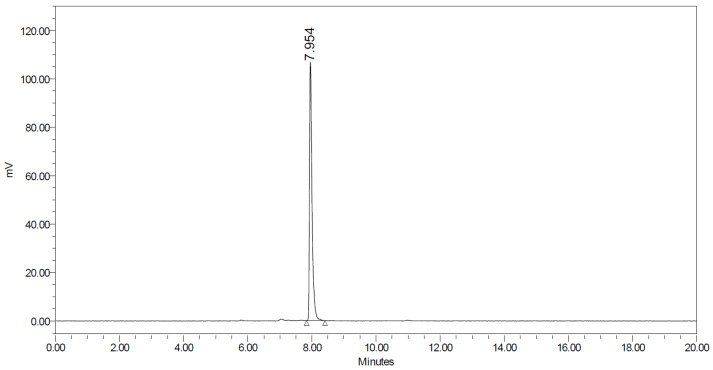
A representative gamma trace of [^18^F]AGAL on analytical HPLC at the end of synthesis showing a single peak at ~8 min.

Procedures for ^18^F-fluorination of pentyne and the subsequent Cu-catalyzed click reaction were adapted from the literature [7,8]. The radiofluorination of tosyl-pentyne occurred via S_N_2 substitution of the tosyl group with [^18^F]KF-K_222_. The fluorinated pentyne was purified via distillation into a second reactor, wherein click reaction with “Intermediate 2” occurred in the presence of copper (II) sulfate, TBTA, and sodium ascorbate. Heat was not required for the click reaction. After a 30 min reaction at ambient temperature (25 °C), the reaction mixture was injected onto a semi-prep HPLC column, and the collected product was further purified on a C18 Plus Light Sep-Pak cartridge (Figure 2 and Figure 3).

**Figure 4:**
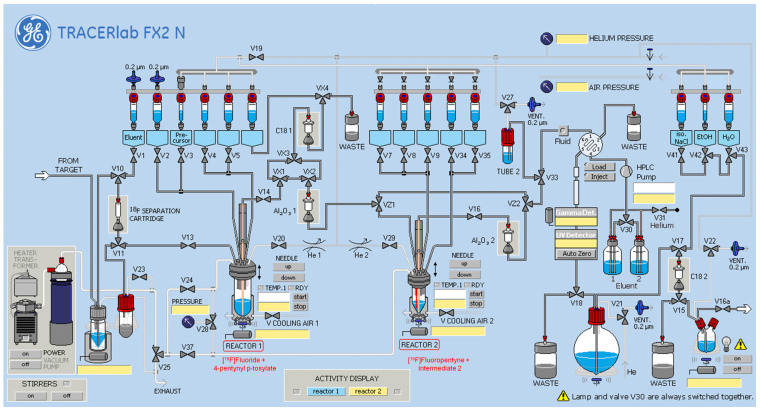
Schematic of the GE FX2 N synthesis module (modified from the manufacturer’s original depiction) with key click-reaction materials shown in Reactor 1 and Reactor 2 for 2-pot radiosynthesis.

Following formulation and sterile filtration, the final product measured 109.9 ± 6.5 mCi (mean ± SD, *n* = 4) in 4.57 ± 0.08 mL of 10% EtOH in saline. Radiochemical purity and molar activity of the product at the end of synthesis, as determined from analytical HPLC, were 96.88 ± 1.13% and 5.73 ± 2.86 Ci/µmol, respectively (Figure 4). The average time required for synthesis and formulation was 76.7 ± 1.9 min.

Stability of the product was monitored via analytical HPLC over time. After 6 h at room temperature, the product showed a decrease in RCP of 5–8%. However, with the addition of sodium ascorbate in the formulation (5 mg/mL), the degradation was significantly reduced and the decrease in RCP of the product was less than 3% after 6 h.

### 2.3. In Vitro Evaluation of AGAL

The AGAL molecule had ≥50% inhibition of specific binding or activity with *IC*_50_ values > 10 µM for a range of human and rodent recombinant receptors and binding sites (e.g., calcium and potassium channels and various subtypes of adrenergic, bombesin, calcitonin, serotonin, acetylcholine, histamine, and glutamate receptors or transporters). This indicates that the AGAL molecule is highly selective with no significant off-target binding.

**Figure 5:**
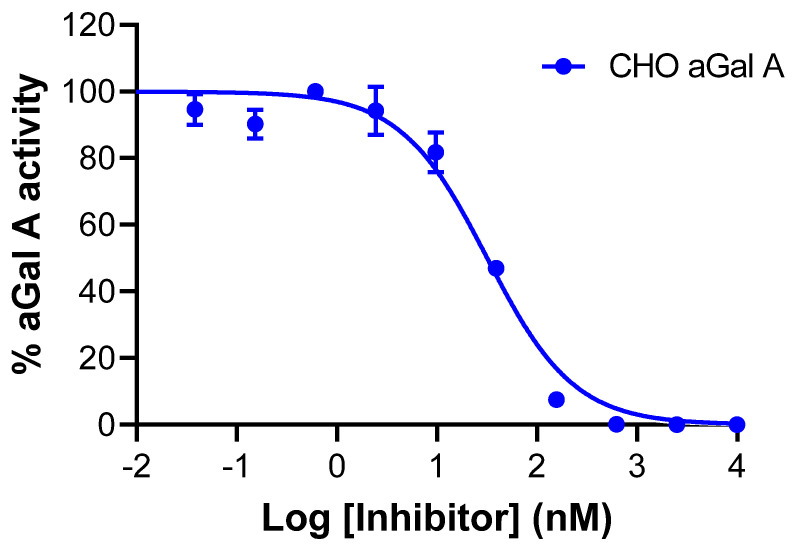
Residual activity of CHO aGal A enzyme measured via hydrolysis of substrate 4−methylumbelliferyl−α−d−galactopyranoside after 30 min of preincubation with AGAL probe.

Using an in vitro affinity assay, measurement of residual activity by preincubating the enzyme with varying concentrations of the probe confirmed that the [^19^F] Bodipy-derivative of the PET tracer acts as a highly potent inhibitor of Chinese hamster ovary cell-derived wild type aGal A (CHO aGal A) with an *IC*_50_ value of 32 nM (Figure 5), which is similar to that reported by Willems et al. [3].

The AGAL molecule showed a LogD value of −0.43. The AGAL molecule has multiple hydroxyl groups contributing to lower lipophilicity, but because of its sugar-like structure it is expected to have improved solubility and cell permeability similar to [^18^F]FDG and other galactosylated prodrugs [9,10].

The microsomal stability results showed that the compound was minimally cleared in human liver microsomes, while being moderately cleared in rat liver microsomes and highly cleared in mouse liver microsomes (Table 1).

The plasma stability results showed that the compound was stable in human plasma but not stable in mouse plasma, with 93% and 18% parent remaining at 30 min post-incubation, respectively. The t_1/2_ in mouse plasma was <10 min (Table 2).

The potential for the compound to reversibly inhibit CYP1A2, 2B6, 2C8, 2C9, 2C19, and 2D6 was investigated by determining the hepatic microsomal metabolism of CYP-selective substrates in the presence (30 μM) and absence of the compound using pooled HLMs (*n* = 200). There was no significant loss of CYP activity at 30 µM compound for all of the CYPs investigated. These results conclude that the compound has low potential to cause CYP-mediated drug–drug interactions (DDIs) by reversible inhibition of metabolism of co-administered drugs.

The bi-directional permeability of 5 µM of the AGAL molecule was assessed in Caco-2 cells. The A-to-B permeability was 0.11 × 10^−6^ cm/s, lower than the *P*_app_ of the low permeable marker nadolol, which had a *P*_app_ value of 0.36 × 10^−6^ cm/s (Table 3). As a result, it is anticipated that the AGAL molecule will be similar to nadolol in that it will have low oral absorption and passive penetration into cells [11,12,13].

In addition to its low permeability, the AGAL molecule also appears to be a substrate of drug efflux transporters as indicated by its efflux ratio of 12.0, which is high (>2.0) and similar to the positive control pevonedistat [14,15] with an efflux ratio of 18.8. To determine the contribution of efflux transporters to this efflux ratio, the bi-directional permeability of the AGAL molecule was assessed in the presence of the P-glycoprotein (P-gp) inhibitor LY335979, the BCRP inhibitor K0143, and the dual inhibitor elacridar. LY335979 reduced the efflux ratio 3-fold, indicating that the AGAL molecule is a substrate of P-gp. K0143 reduced the efflux ratio by 1.4-fold and does not meet the recommended criteria (2-fold reduction in efflux ratio) for the AGAL molecule to be considered a substrate of BCRP (FDA 2020, ICH 2022). LY335979 and elacridar reduced the efflux ratio by 67 and 76 percent, respectively, indicating that P-gp mediates most of the active efflux of the AGAL molecule by Caco-2 cells. However, neither inhibitor completely reduced the efflux ratio to 1, indicating that other efflux transporters may be involved in the active efflux of this molecule. Overall, the results demonstrate that P-gp is a potential liability in the absorption of the AGAL molecule by the gut or blood brain barrier [16].

### 2.4. In Vivo PET/CT Imaging

**Figure 6:**
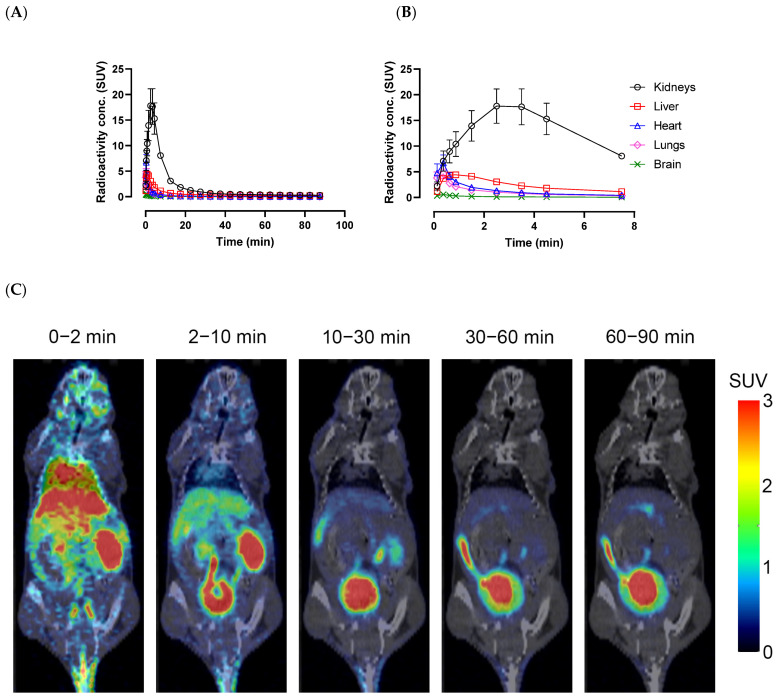
Dynamic in vivo PET/CT imaging in wild type mice after [^18^F]AGAL intravenous injection. Time course of average radioactivity concentrations (*n* = 4, Mean ± SD) from various organs shown for full 90 min (**A**) and first 10 min (**B**) after tracer injection. (**C**) Representative coronal sections of PET/CT scan images from a wild type mouse with frames averaged between 0–2 min, 2–10 min, 10–30 min, 30–60 min, and 60–90 min are shown. SUV: Standardized uptake value.

After intravenous injection of [^18^F]AGAL in wild type mice, the radioactivity rapidly accumulated in key organs such as the kidneys, liver, and heart, with the kidneys having the highest peak standardized uptake value (SUV ~18) at 3 min followed by rapid washout from all organs to lower than 1 SUV by 30 min and remaining low until the end of the 90 min scan (Figure 6A,B). Representative dynamic PET images show that the initial rapid uptake is due to blood pool uptake and higher kidney uptake, with later high urinary bladder uptake indicating quick elimination by renal clearance. No bone uptake was noted in the images, indicating absence of tracer defluorination (Figure 6C).

## 3. Materials and Methods

### 3.1. Radiolabeling of AGAL

Approximately 2000 mCi of [^18^F]fluoride was produced on a GE PETtrace 800 cyclotron and delivered to the ^18^F target delivery vial of a GE FX2 N radiosynthesis module (Figure 4). The irradiated target water was passed through a pre-conditioned Sep-PAK Light QMA Cartridge to trap [^18^F]fluoride, followed by elution from the cartridge into reactor 1 using 3 mg K_2_CO_2_ and 13 mg Cryptand 222 in 0.6 mL of 50% acetonitrile in water. The contents of reactor 1 were dried via azeotropic distillation using a combination of heating, helium flow, and vacuum. An additional 1 mL of acetonitrile was introduced to reactor 1 and the azeotropic drying process was repeated. The reactor temperature was then reduced to 30 °C, followed by introduction of 4-pentynyl p-tosylate (10 mg, ABX GmbH, Radeberg, Germany) in 1.0 mL acetonitrile (anhydrous). The reactor was then sealed and heated to 110 °C and allowed to react with stirring for 3 min. Reactor 1 was then opened to reactor 2, with the reactor 1 needle in the “up” position. Helium was then introduced to reactor 1 to induce distillation from reactor 1 to reactor 2, where the distillate (^18^F-fluoropentyne) was bubbled through a line submerged in a room temperature (25 °C) solution preloaded into reactor 2 during module set-up (300 μL 0.1 M CuSO_4_ in water, 40 mg sodium L-ascorbate, 15 mg TBTA, 100 μL methanol, 100 μL *N*,*N*-dimethylformamide, and 0.5 mg of “Intermediate 2” in 50 µL acetonitrile). The distillation from reactor 1 was allowed to continue until the activity in reactor 2 showed no further increase—approximately 1–2 min—with activity of the distillate in reactor 2 ranging between 900–1050 mCi. Reactor 2 was then isolated and stirred at room temperature for 30 min. The contents of reactor 2 were then transferred to the HPLC pre-loading vial. Reactor 2 was then rinsed with 2 mL of water, which was subsequently transferred to the HPLC pre-loading vial. The combined crude mixture in the HPLC pre-loading vial was loaded onto a 5 mL HPLC loop which had been previously filled with semi-preparative HPLC mobile phase (22% acetonitrile in H_2_O) to minimize injection of air onto the HPLC column. The crude mixture was purified on a semi-preparative HPLC column (Phenomenex Luna 5 µm, C18(2), 100 A and 250 × 10 mm, 00G-4252-N0) with 22% acetonitrile in water as the mobile phase (flow rate = 3.5 mL/min). The product peak (~11–14 min) was collected into a round-bottom flask containing 30 mL sterile water. The contents of the round-bottom flask were passed through a preconditioned C18 Plus Light Sep-PAK cartridge, where ^18^F-AGAL was trapped. The cartridge was washed with 10 mL water (USP, sterile for irrigation), followed by elution of ^18^F-AGAL to the FX2 N product vial using 0.5 mL ethanol. A 4.5-mL aliquot of 0.9% normal saline for injection was then passed through the Sep-PAK cartridge and into the product vial. The contents of the product vial were transferred through a 0.2 μm air-eliminating filter and into a 10 mL sterile empty vial fitted with a filtered-vent needle. To determine the stability of the final product, analytical HPLC injections were made at 2, 4, and 6 h post-synthesis (Waters XSelect CSH C18 3.5 μm, 150 × 4.6 mm, 186,005,270; A = H_2_O, B = CH_3_CN; 0 min, 10% B—1.5 min, 10% B—6.0 min, 30% B—9.0 min, 35% B—13.0 min, 90% B—15.0 min, 90% B—17.0 min, 10% B—20 min, and 10% B). An aliquot of the product was also formulated with sodium ascorbate (5 mg/mL in normal saline, pH 5), and stability data were compared to that of the product formulated in saline alone.

### 3.2. In Vitro Testing of AGAL for Selectivity and Affinity

The AGAL molecule was tested for estimated inhibition potency (*IC*_50_) and/or inhibition constants (*K*_i_) on 167 protein targets (Eurofins SpectrumScreen).

The *IC*_50_ assessment for the aGal inhibitors was performed using the fluorescent substrate in a McIlvaine buffer at pH 4.6. The CHO aGal A enzyme at 1 nM was pre-incubated with different concentrations of the inhibitor or DMSO as control for 30 min at room temperature. The fluorescent substrate 4-methylumbelliferyl-α-d-galactopyranoside (Research Products International company, catalog number M65400, Mount Prospect, IL, USA) was then added to a final concentration of 5 mM. The reaction was quenched with 0.5 M sodium bicarbonate buffer, with pH 10.5, and plates were read using a SpectraMax reader at excitation and emission wavelengths of 360 and 460 nm, respectively. Data were corrected for background fluorescence and *IC*_50_ calculation was performed using an inhibitory dose–response curve fitting in GraphPad Prizm 8.0.

### 3.3. LogD Determination of AGAL Molecule

The distribution of AGAL between an organic solvent (octanol) and aqueous buffer (pH 7.4) was tested using the miniaturized shake flask method in octanol/buffer (1:1). This was accomplished by spiking the test compound (prepared in DMSO) into pre-equilibrated octanol/buffer phases at a concentration of 66.6 μM followed by shaking at 880 rpm for 1 h. The concentration of the test compound in both phases was analyzed using LC–MS/MS.

### 3.4. In Vitro Liver Microsomal Metabolism Study

Metabolic stability was assessed by incubating compounds in the presence of human, rat, and mouse liver microsomal fractions (Xenotech, Kansas City, KS, USA). All chemicals and reagents, including control compounds, were purchased from commercial sources such as Sigma-Aldrich (St. Louis, MO, USA) or Toronto Research Chemicals (North York, ON, Canada). Liver microsomal incubations were conducted with 100 mM phosphate buffer, 0.5 mg/mL of liver microsomal protein, 2 mM NADPH, 2.5 mM UDPGA, 3 mM MgCl_2_, alamethicin, and 1 μM test article for a total incubation volume of 80 µL. All reagents except the liver microsomes were mixed together and aliquoted (60 μL) into 96-well plates. The reaction was initiated by adding the microsomes (20 μL). At 0, 3, 7, 12, 20, and 30 min time points, the reactions were terminated with the addition of acetonitrile-containing carbutamide (as an internal standard). The terminated samples were then centrifuged at 2 °C to pellet the protein and the supernatant was injected onto an LC–MS. The test article was monitored for loss over time and the t_1/2_ was determined. The calculated t_1/2_ was then used as described below to determine the E_h_ (hepatic extraction ratio) for both human and rat.

The intrinsic clearance (CL_int_), hepatic clearance (CL_h_), and hepatic extraction ratio (E_h_) were calculated using the equations below [17,18,19].

CL_int_ = (0.693/t_1/2_ in vitro) × (mL incubation volume/mg microsomal protein) × (X mg of microsomal protein/gram of liver) × (Y g of liver/kg of body weight)(1)

CL_h_ = Q × CL_int_/Q + CL_int_ (2)

E_h_ = CL_h_/Q (3)

where:

CL_int_ = intrinsic clearance (calculated from microsomal incubations)
X = 40 for human, 41.5 for rat and 45 for mouse
Y = 21.4 for human, 40 for rat and 50 for mouse
t_1/2_ = half-life
CL_h_ = hepatic clearance
Q = liver blood flow rate = 1.24 L/h/kg for human, 4.8 L/h/kg for rat, and 5.4 L/h/kg for mouse
E_h_ = hepatic extraction ratio
CL_h_ = hepatic clearance

### 3.5. Plasma Stability Study

Briefly, plasma stability was assessed by incubating compounds in the presence of human (pooled) and mouse (pooled) plasma (Bioreclamation, Westbury, NY, USA). All chemicals and reagents including control compounds were purchased from commercial sources such as Sigma-Aldrich or Toronto Research Chemicals. Plasma incubations were conducted by placing plasma (95 μL) in a 96-well microtiter plate and warming to 37 °C for 5 min. The reaction was initiated with the addition of the test article (5 μL of 20 μM), totaling 100 μL in incubation volume. At 0, 15, 30, 60, and 120 min time points, the reactions were terminated with the addition of acetonitrile-containing carbutamide (as an internal standard). The terminated samples were then centrifuged at 2 °C to pellet the protein, and the supernatant was injected onto an LC–MS. The test article was monitored for loss over time and the t_1/2_ was determined.

### 3.6. Cytochrome 450 Enzyme Inhibition Assays

All chemicals and reagents including control compounds were purchased from commercial sources such as Sigma-Aldrich or Toronto Research Chemicals. The test article was tested for reversible inhibition of CYPs in human liver microsomes (HLM) (0.2 mg/mL protein) at a concentration range of 0–30 μM. Each condition was incubated in duplicate. The test article (0–30 μM), NADPH, and the CYP-selective probe substrate were incubated in a 96-well plate at 37 °C in 0.1 M potassium phosphate buffer, pH 7.4. The reactions were initiated by adding the HLMs. The total reaction volume was 100 μL. The CYP-selective probe substrates used, and their concentrations, are listed in Table 4.

After the incubation period (10 min for CYP1A2, 2B6, and 2C9; 15 min for CYP2C19; and 4 min for CYP2C8 and 2D6 to accommodate linear kinetic conditions) at 37 °C, the reactions were terminated by the addition of 100 μL of ice-cold acetonitrile-containing carbutamide as an IS, then centrifuged at 2500× *g* for 10 min at 2 °C. A 150 μL aliquot of supernatant for each CYP assay, except for CYP1A2, was then transferred to a new plate for analysis. For CYP1A2, 80 μL of supernatant was transferred to the plate and further diluted with 80 μL of water before analysis. All samples were analyzed with liquid chromatography with tandem mass spectrometry (LC–MS/MS).

### 3.7. AGAL Cell Permeability Studies

#### 3.7.1. Caco-2 Cell Culture

The Caco-2 cell culture and bidirectional permeability assays used in the study were adapted from previous studies at Takeda [20,21]. Briefly, Caco-2 cells were obtained from American Type Culture Collection (ATCC) (Manassas, VA, USA) and were cultured in 175 cm^2^ tissue culture T-flasks (Corning Life Sciences, Corning, NY, USA). For bidirectional permeability assays, cells were plated onto Costar 24-well culture plates (0.33 cm^2^/well, 0.4 mm pore size (Corning Life Sciences, Corning, NY, USA) by adding 1 × 10^5^ cells suspended in 0.2 mL of culture medium (Dulbecco’s modified Eagle’s medium [DMEM] with 0.1 mM non-essential amino acids, 2 mM L-glutamine, 4.5 g/L glucose, and 10% fetal bovine serum [FBS]) to the upper chamber of each well of the 24-well transwell plate. Cell-free culture medium (40 mL) was added to the lower chamber reservoir (Corning Life Sciences, Corning, NY, USA). The transwell plates were then incubated at 37 °C in an atmosphere of 5% CO_2_ for 25 days to ensure complete enterocyte-like differentiation of the cells, and the culture medium was changed every three to four days during the incubation period. Cell monolayers with transepithelial electrical resistance (TEER) values > 250 ohms × cm^2^ were used for the bidirectional permeability assays.

#### 3.7.2. Bidirectional Permeability Studies

All chemicals and reagents including control compounds were purchased from commercial sources such as Sigma-Aldrich or synthesized in house (pevonedistat and LY335979). Bidirectional transport studies were performed at 37 °C. Before each experiment, the confluent cell monolayers on transwell inserts were washed and equilibrated for 30 min with transport media (Hank’s Balanced Salt Solution [HBSS] containing 25 mM 4-(2-hydroxyethyl)-1-piperazineethanesulfonic acid [HEPES] and 10 mM glucose, pH 7.4 or 6.5). The bidirectional permeability of 5 µM of the cyclohexane derivative was assessed alone or in the presence of 5 µM LY335979, 5 µM K0143, or 10 µM Elacridar to inhibit P-gp, BCRP, and both efflux transporters, respectively. Additionally, the bi-directional permeability of 5 µM solutions of nadolol (low permeable control marker), minoxidil (high permeable marker), propranolol (high permeable marker), and pevonedistat (efflux transporter control) was also assessed. The experiment was initiated by adding a solution containing the test compounds and LY335979 to either the apical (for apical-to-basolateral [A-to-B] transport) or basolateral (for basolateral-to-apical [B-to-A] transport) compartment. Lucifer yellow (50 µM) was added to each donor chamber in the experiment to serve as a paracellular transport control and to monitor cell monolayer integrity. The apical volume was 0.2 mL and the basolateral volume was 1.0 mL. Bovine serum albumin (BSA, Sigma Aldrich) (5%) was added to the transport media on the receiver side to prevent non-specific binding of the test compound to the polystyrene well. After the addition of test compound into the donor side, 120 mL aliquots of the receiving solution were withdrawn from the receiver side at 30 and 60 min. A 120 mL aliquot of fresh transport media was immediately added to the receiver side at 30 and 60 min. The samples were placed into 96-well black plates (PerkinElmer Life Sciences, Boston, MA, USA) for fluorescence measurement (excitation at 450 nm and emission at 535 nm). After this measurement, the amounts of cyclohexane derivative and control compounds in each sample were quantified with liquid chromatography–mass spectrometry (LC–MS). The time course of drug transported across cell monolayers was analyzed to determine the time and rate of steady-state flux. The transport rate (total amount of drug present in the receiver chamber per unit time), *P*_app_, and efflux ratio were calculated as described in Equations (4) and (5).

*P*_app_ = (*dQ/dt*)/(A × C_0_) (4)

where:

*dQ/dt* = total amount of drug present in the receiver chamber per unit time (nmol/s)
A = surface area (cm^2^, in the present studies A= 0.33 cm^2^)
C_0_ = initial drug concentration in the donor chamber (nmol/mL)
*P*_app_= apparent permeability (cm/s × 10^−6^).

Efflux ratio (B: A) = *P*_app_, B–to–A: *P*_app_, A–to–B (5)

where:

*P*_app_, A−to−B = *P*_app_ calculated where the apical side is the donor, and the basolateral side is the receiver
*P*_app_, B−to−A = *P*_app_ calculated where the basolateral side is the donor, and the apical side is the receiver.

All experiments were performed in triplicate.

### 3.8. In Vivo PET/CT IMAGING

[^18^F]AGAL PET imaging was performed on a dedicated small animal PET/CT scanner (Siemens Multimodality Inveon, Siemens Medical Solutions USA Inc., Knoxville, TN, USA). The wild-type mice (*n* = 3, male 129S6) were anesthetized with 3% isoflurane/medical air inhalation prior to the radiotracer injection and throughout the scan duration. A bolus intravenous injection (via the lateral tail vein) of [^18^F]AGAL (~7.4 MBq) was administered followed by dynamic PET scans in list mode for 90 min. After the PET acquisition, a low-dose CT scan was acquired (80 kVp, 0.5 mA) for anatomical reference and to provide guidance for the delineation of selected tissues volume of interest (VOI). The acquired PET data were sorted into 0.5 mm sinogram bins and 25 time frames (4 × 15 s, 4 × 60 s, and 17 × 300 s) for image reconstruction using FORE/3D-OSEM-MAP. The reconstructed PET/CT images were analyzed with the Siemens Inveon Research Workplace software v4.0 or other appropriate software. The radioactivity retention within the selected tissue was obtained from mean voxel intensity values within the VOI and then converted to megabecquerels per milliliter using the calibration factor determined for the Inveon PET System. These values were then divided by the administered activity in megabecquerels and animal body weight to obtain an image VOI-derived standardized uptake value (SUV).

## 4. Conclusions

The present study showed a successful radiosynthesis of a new PET probe [^18^F]AGAL targeting alpha-galactosidase A enzyme, and its evaluation in in vitro cell assays demonstrated highly specific and selective binding, with favorable pharmacokinetic properties in human compared to mouse plasma and microsomes. A pilot in vivo imaging study demonstrated rapid clearance from the various organs in wild type mice, thus providing low background signal in vivo. Additional studies in a mouse model of Fabry disease with and without gene or enzyme replacement therapies are warranted to further validate this probe for in vivo use in therapeutic imaging trials of Fabry disease.

## Figures and Tables

**Table 1:** In vitro metabolism study in liver microsomes of different species.

Sample	Human			Rat			Mouse		
	CL_int_ (L/h/Kg)	CL_h_ (L/h/Kg)	E_h_	CL_int_ (L/h/Kg)	CL_h_ (L/h/Kg)	E_h_	CL_int_ (L/h/Kg)	CL_h_ (L/h/Kg)	E_h_
AGAL	<0.363	<0.281	<0.226	10.9	3.34	0.695	17.6	4.13	0.766
Dextromethorphan	2.59	0.838	0.676	38.5	4.27	0.889	33.1	4.64	0.860

CL_int_: Intrinsic clearance; CL_h_: Hepatic clearance; E_h_: Hepatic Extraction ratio.

**Table 2:** In vitro plasma stability study in human and mouse plasma.

Sample	Plasma Half-Life, t_1/2_ (Min)	
	Human	Mouse
AGAL	208	9.83
Enalapril (Positive Control)	283	30.6
Propantheline (Positive Control)	7.22	26

**Table 3:** Apparent permeability of AGAL in Caco-2 cells.

Sample	*P*_app_, A-to-B (×10^−6^ cm/s)		*P*_app_, B-to-A (×10^−6^ cm/s)		Ratio (B/A)
	Mean	SEM	Mean	SEM	
Pevonedistat (positive control for P-gp)	3.37	0.42	63.4	3.3	18.8
Nadolol (low permeability control)	0.36	0.02	1.45	0.03	4.1
Propranolol (high permeability control)	43.7	4.8	44.4	0.98	1
Minoxidil (medium permeability control)	7.81	0.64	9.78	0.48	1.3
AGAL	0.11	0.02	1.36	0.04	12
AGAL + LY335979 (P-gp inhibitor)	0.25	0.04	0.95	0.01	3.9
AGAL + KO143 (BCRP inhibitor)	0.15	0	1.31	0.01	8.7
AGAL + Elacridar (GF918) (dual inhibitor of P-gp and BCRP)	0.25	0.03	0.74	0.07	2.9

SEM: Standard error of mean.

**Table 4:** Cytochrome P450 Enzyme Substrates and Final Concentrations for Direct Inhibition Assay.

CYP	Substrate	Final Incubation Concentrations (µM)
1A2	Phenacetin	50
2B6	Bupropion	100
2C8	Amodiaquine	2
2C9	Diclofenac	5
2C19	S-mephenytoin	50
2D6	Dextromethorphan	5

CYP: cytochrome P450 enzyme.

## Data Availability

All data are contained within the article.
